# Application of a novel swallowable telemetric device for real-time luminal blood detection to guide timing of enteroscopy in a patient with occult GI bleeding: a case report

**DOI:** 10.1016/j.igie.2023.11.009

**Published:** 2023-11-29

**Authors:** Joachim Rainer, Paolo Biancheri, Giuliano Francesco Bonura, Simona Deiana, Tommaso Gabbani, Noemi Gualandi, Roberta Pileggi, Paola Soriani, Mauro Manno

**Affiliations:** Azienda Unità Sanitaria Locale di Modena, Carpi, Emilia Romagna, Italy

Small-bowel bleeding should be suspected when results of an initial diagnostic workup with EGD and colonoscopy are negative yet clinical evidence for GI blood loss is present, either in the form of visible intestinal hemorrhage (“overt”) or iron-deficiency anemia (IDA) without evidence of an extraintestinal bleeding site (“occult”).

Although overt small-bowel bleeding events generally show more acute clinical manifestations, and small-bowel examination modalities carry higher diagnostic yield,^1^ the origin of occult small-bowel bleeding can be difficult to assess.

Due to this distinction, in occult-suspected small-bowel bleeding, the less invasive test of video capsule–assisted enteroscopy (VCE) is recommended per recent guidelines.^2^^,^^3^

Furthermore, even if a potential bleeding site is identified on VCE, the less acute nature of IDA opens up different therapeutic strategies, as the decision to perform device-assisted enteroscopy (DAE) hinges on the severity of anemia and the response to conservative treatment, as well as the patient’s performance status and adverse event risk.

In the current case, the decision-making process to perform DAE in a frail patient was aided by a novel diagnostic tool called HemoPill (Ovesco, Tübingen, Germany). HemoPill is a swallowable capsule 26.3 × 7.0 mm in size; it can monitor in real time for the presence of blood during its transit through the GI tract by assessing the optical characteristics of the material in its measuring window. The capsule is calibrated to display a value of 0 in the absence of any material in the measuring gap and a value ≥1.0 in the presence of blood or hematin. Hence, values ≥1.0 are interpreted as a “positive“ test result in all cases, whereas, if registered within 10 minutes after capsule ingestion, even values ≥0.8 are deemed “positive.“ Due to this photoabsorption-based approach and contrary to other capsule-based techniques, use of the HemoPill does not require bowel preparation.^4^

## Case presentation

We report on a 71-year-old male patient who presented at our emergency department in January 2022 with fatigue and shortness of breath. On laboratory examinations, IDA (hemoglobin 8.0 g/dl, hematocrit 26.5%, mean corpuscular volume 67.7 fl, mean corpuscular hemoglobin 20.3 pg, and ferritin 14 ng/mL) was evident. The patient denied overt blood loss.

The patient’s medical history was already extensive, with known ischemic heart insufficiency, peripheral artery disease, chronic obstructive pulmonary disease, HIV, and hepatitis C virus infection, as well as a recent diagnosis of Kaposi‘s sarcoma. Due to a complex coronary artery disease, dual antiplatelet therapy with aspirin and ticagrelor was part of his medication.

The patient was initially stabilized with the use of blood transfusions. Results of upper and lower GI endoscopy were both negative for potential bleeding sites.

**Figure 1:**
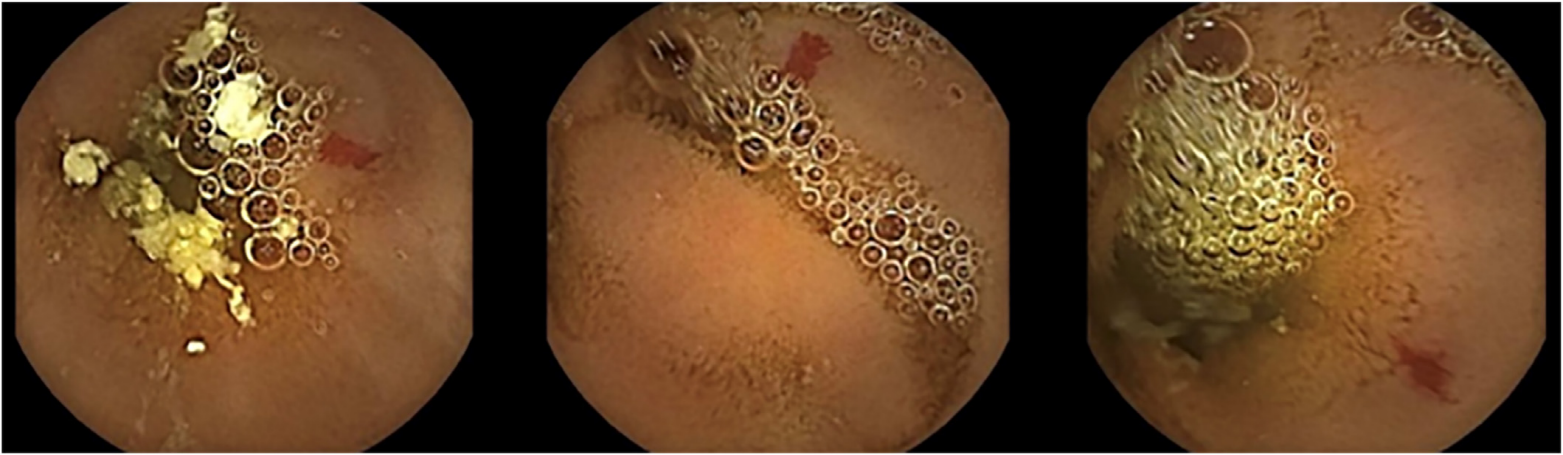
Video capsule–assisted enteroscopy findings of angiodysplastic lesions (type 1b according to Yano-Yamamoto classification) of the proximal jejunum.

On subsequent VCE examination, small-bowel transit time was 2 hours 10 minutes. Within 1 to 13 minutes from pylorus passage, evidence of 3 small lesions suspect for angiodysplasia (5 mm in diameter, type 1b according to Yano-Yamamoto classification) was noted, with no signs of active bleeding during registration (Fig. 1).

During his hospital stay, the patient was diagnosed with an intercurrent and asymptomatic SARS-CoV-2 infection. Due to his temporarily stable hemoglobin level, a decision was made to delay DAE, and the patient was discharged with oral iron supplementation.

After 6 weeks, the patient presented himself at our emergency department with symptoms of congestive heart failure, hypoxemic respiratory insufficiency, and recurrent IDA (hemoglobin 8.7 g/dL). He was readmitted, and diuretic treatment regimen as well as endovenous iron supplementation were initiated.

**Figure 2:**
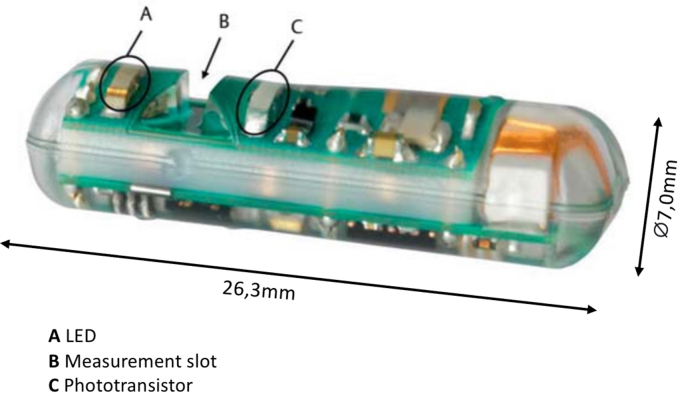
HemoPill capsule.

The decision was made not to repeat VCE and instead administer the HemoPill (Fig. 2) to confirm that the previously identified jejunal lesions were actively bleeding.

## Results

The measurement revealed a HemoPill index within normal range in the first hour after ingestion and a peak HemoPill index of 1.0 (reference value <0.8 during first 10 minutes after ingestion, <1.0 subsequently) at 1 hour 47 minutes after capsule administration. This finding was in keeping with small-bowel blood loss and therefore confirmed the VCE findings of the previous hospital stay.

**Figure 3:**
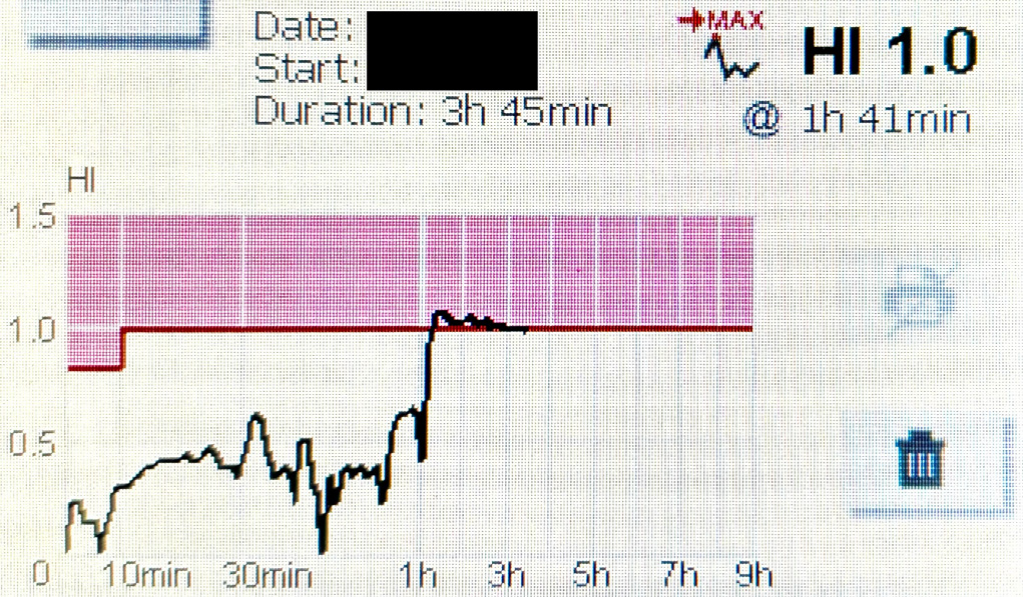
Result of HemoPill measurement.

Figure 3 shows the signal registration of the HemoPill monitor.

On DAE, performed with the patient under general anesthesia with a spiral enteroscopy technique and antegrade approach, the lesions were identified in the proximal jejunum as type Ib according to the Yano-Yamamoto classification; they were successfully treated by using thermal therapy, applying argon plasma coagulation. The patient was discharged the following day.

After 1 year of follow-up, the patient is well and has not had any further anemia recurrence.

## Discussion

In this case report, a novel technique of intraluminal blood detection was used and was able to confirm that DAE would be beneficial for a frail patient with occult small-bowel bleeding.

DAE is generally performed with the patient in deep sedation or under general anesthesia, and the risk for adverse events, although fairly low in the general population, is elevated in therapeutic DAE^5^ and may be elevated in patients with multiple health conditions.^6^ In IDA, the therapeutic alternative of iron supplementation and occasional blood transfusion is oftentimes preferred by therapists. On the other hand, in patients with chronic heart failure, IDA correlates with cardiac disease–related mortality^7^ and should therefore be avoided, and its causes should be treated.^8^ Moreover, the beneficial effect of long-term dual antiplatelet therapy for severe coronary artery disease is often withheld due to the risk of bleeding. In our view, the risks and potential benefits of DAE in frail individuals with occult small-bowel bleeding should be carefully assessed and discussed with patients.

In this specific clinical scenario, a previous VCE diagnosis of jejunal angioectasias was made; however, the VCE had been performed 2 months earlier. As routinely experienced during clinical practice, small-bowel lesions may be intermittently bleeding; hence, it is not automatic that bleeding lesions on VCE can be identified and treated with subsequent DAE, especially in the absence of overt clinical signs of bleeding. In our case, we wanted to check for the presence of blood in the small bowel with a noninvasive test given the patient’s severe frailty and respiratory status; we hypothesized that the result of the HemoPill monitoring would inform our subsequent decision to perform DAE. Indeed, we had discussed with the patient and his family the alternative option of conservative management by iron supplementation and blood transfusions in case the HemoPill measurements were negative, especially as American Society of Anesthesiologists class IV patients may face significant risks undergoing DAE in deep sedation.

Although the HemoPill has been developed primarily as a tool to triage patients with suspected upper GI bleeding upon presentation in the emergency department,^9^ it may prove to be an aid to decision-making in a variety of clinical settings when GI blood loss is suspected, including in occult-suspected small-bowel bleeding. However, data on its clinical use are currently lacking, and prospective studies are needed. Our case report suggests that the accuracy of the HemoPill to predict clinical outcomes and need for intervention should be further evaluated, both in the field of suspected upper GI bleeding, as well as in suspected small-bowel bleeding.

In conclusion, our case illustrates how a swallowable telemetric device for luminal blood detection can aid clinical decision-making in complex patients. We encourage further studies to evaluate whether this device can predict clinical outcomes and the need for hemostatic treatment in subsequent endoscopy.

## Disclosure

All authors disclosed no financial relationships.
